# The 100 Top-Cited Studies on Dyslexia Research: A Bibliometric Analysis

**DOI:** 10.3389/fpsyt.2021.714627

**Published:** 2021-07-22

**Authors:** Shijie Zhang, Hong Fan, Yonggang Zhang

**Affiliations:** ^1^Department of Respiratory and Critical Care Medicine, West China Hospital/West China School of Medicine, Sichuan University, Chengdu, China; ^2^Department of Periodical Press and National Clinical Research Center for Geriatrics, West China Hospital, Sichuan University, Chengdu, China; ^3^Chinese Evidence-Based Medicine Center, West China Hospital, Sichuan University, Chengdu, China

**Keywords:** dyslexia, bibliometric analysis, top-cited, citation analysis, citation

## Abstract

**Background:** Citation analysis is a type of quantitative and bibliometric analytic method designed to rank papers based on their citation counts. Over the last few decades, the research on dyslexia has made some progress which helps us to assess this disease, but a citation analysis on dyslexia that reflects these advances is lacking.

**Methods:** A retrospective bibliometric analysis was performed using the Web of Science Core Collection database. The 100 top-cited studies on dyslexia were retrieved after reviewing abstracts or full-texts to May 20th, 2021. Data from the 100 top-cited studies were subsequently extracted and analyzed.

**Results:** The 100 top-cited studies on dyslexia were cited between 245 to 1,456 times, with a median citation count of 345. These studies were published in 50 different journals, with the “Proceedings of the National Academy of Sciences of the United States of America” having published the most (*n* = 10). The studies were published between 1973 and 2012 and the most prolific year in terms of number of publications was 2000. Eleven countries contributed to the 100 top-cited studies, and nearly 75% articles were either from the USA (*n* = 53) or United Kingdom (*n* = 21). Eighteen researchers published at least two different studies of the 100 top-cited list as the first author. Furthermore, 71 studies were published as an original research article, 28 studies were review articles, and one study was published as an editorial material. Finally, “Psychology” was the most frequent study category.

**Conclusions:** This analysis provides a better understanding on dyslexia and may help doctors, researchers, and stakeholders to achieve a more comprehensive understanding of classic studies, new discoveries, and trends regarding this research field, thus promoting ideas for future investigation.

## Introduction

Dyslexia is a common learning disorder that affects between 4 and 8% of children (1–3), and often persists into adulthood (4, 5). This neurodevelopmental disorder is characterized by reading and spelling impairments that develop in a context of normal intelligence, educational opportunities, and perceptual abilities (4, 6). Reading and spelling abilities can be affected together or separately. The learning abilities of children with dyslexia are significantly lower than those of their unaffected pairs of the same age. Generally, difficulties begin to show during the early school years. Dyslexia is a complex multifactorial disorder whose etiology has not been fully elucidated, and it has caused great social and economic burdens. Over the last few decades, the research on dyslexia has made some progress. For example, some studies have shown that dyslexia has a strong genetic background that can affect brain anatomy (7, 8) and function (9, 10). But a citation analysis on dyslexia that reflects these advances is lacking.

The publication of study results in scientific journals is the most effective strategy to disseminate new research findings. A high number of citations can indicate the potential of a paper to influence the research community and to generate meaningful changes in clinical practice (11). Citation analysis is a type of quantitative and bibliometric analytic method designed to rank papers based on their citation counts. The latest and up-to-date research findings on dyslexia are well-reflected in recent scientific papers (12), particularly in the most cited ones (13, 14). By analyzing the most cited studies, especially the 100 top-cited studies, we can gain better insight into the most significant advances made in the field of dyslexia research over the course of the past several decades (15). This retrospective bibliometric approach has been used for many other diseases, such as diabetes (16), endodontics (17), cancer (18). However, to date, no bibliometric analyses have been conducted in the field of dyslexia. Therefore, the aim of the present study was to analyze the 100 top-cited studies in the field of dyslexia.

## Materials and Methods

### Search Method and Inclusion Criteria

This retrospective bibliometric analysis was conducted using the Web of Science Core Collection database. The Web of Science Core Collection is a multidisciplinary database with searchable authors and abstracts covering a vast science journal literature (19). It indexes the major journals of more than 170 subject categories, providing access to retrospective data between 1945 and the present (20). On May 20th, 2021, we conducted an exhaustive literature retrieval, regardless of the country of origin, publication year, and language. The only search term used was “dyslexia” and the search results were sorted by the number of citations.

### Article Selection

Two authors independently screened the abstracts or full-texts to identify the 100 top-cited articles about dyslexia. Disagreements were resolved through discussion. Only studies that focused on dyslexia were included in subsequent analyses. Studies that only mentioned dyslexia in passing were excluded.

### Data Extraction

The final list of the 100 top-cited studies on dyslexia was determined by total article citation counts. We extracted the following data for each article: title, authors, journal, language, total citation count, publication year, country, journal impact factor, type of article, and Web of Science subject category. If the reprint author had two or more affiliations from different countries, we used the first affiliation as the country of origin. If one article was listed in more than one subject category, the first category was selected. If one article had more than one author, we selected the first-ranked author as the first author and the last-ranked author as the last-author.

### Data Analysis

SPSS 11.0 (Chicago, IL, USA) was used to count the frequency. We analyzed the following data: citation count, year of publication, country, the first author, journal, language, type of study, and Web of Science subject category.

## Results

### Citation Analysis

The 100 top-cited studies on dyslexia based on total citations are listed in Table 1. The total citation count for these 100 articles combined was 42,222. The total citation count of per study ranged from 245 to 1,456 times, with a median citation count of 345. Only 3 studies were cited more than 1,000 times, and the rest of the studies were cited between 100 and 1,000 times. The title of the top-cited study, which also had the largest mean citation per year count (*n* = 91), was “Reading acquisition, developmental dyslexia, and skilled reading across languages: a psycholinguistic grain size theory,” which was published by Ziegler et al. in Psychological Bulletin in 2005 (21). The second top-cited study, which also had the second-highest mean citation per year count (*n* = 80), was published by Vellutino et al. (22). In addition, we also identified the 100 top-cited studies on dyslexia based on mean citation per year, whose results were shown in Supplementary Table 1.

**Table 1:** The 100 top-cited studies on dyslexia based on total citations.

**Ranking**	**Title**	**Journal**	**Total citation**	**Publication year**	**Mean citation per year**	**Country**	Impact factor in the year of publication^*^
1	Reading acquisition, developmental dyslexia, and skilled reading across languages: a psycholinguistic grain size theory	Psychological Bulletin	1,456	2005	91	France	9.746
2	Specific reading disability (dyslexia): what have we learned in the past four decades?	Journal of Child Psychology and Psychiatry	1,359	2004	80	USA	2.782
3	The double-deficit hypothesis for the developmental dyslexias	Journal of Educational Psychology	1,048	1999	48	USA	1.893
4	Rapid automatized naming (ran)—dyslexia differentiated from other learning-disabilities	Neuropsychologia	970	1976	22	USA	N/A
5	Developmental dyslexia–4 consecutive patients with cortical anomalies	Annals of Neurology	955	1985	27	USA	N/A
6	A definition of dyslexia	Annals of Dyslexia	863	2003	48	USA	1.261
7	Theories of developmental dyslexia: insights from a multiple case study of dyslexic adults	Brain	832	2003	46	France	7.967
8	To see but not to read; the magnocellular theory of dyslexia	Trends in Neurosciences	724	1997	30	UK	17.084
9	Developmental dyslexia and specific language impairment: same or different?	Psychological Bulletin	698	2004	41	UK	7.701
10	Physiological and anatomical evidence for a magnocellular defect in developmental dyslexia	Proceedings of the National Academy of Sciences of the United States of America	668	1991	22	USA	N/A
11	Dyslexia: cultural diversity and biological unity	Science	665	2001	33	Italy	23.329
12	Varieties of developmental dyslexia	Cognition	635	1993	23	Australia	N/A
13	Disruption of posterior brain systems for reading in children with developmental dyslexia	Biological Psychiatry	631	2002	33	USA	5.915
14	Phonology, reading acquisition, and dyslexia: insights from connectionist models	Psychological Review	598	1999	27	USA	6.803
15	Functional disruption in the organization of the brain for reading in dyslexia	Proceedings of the National Academy of Sciences of the United States of America	598	1998	26	USA	9.821
16	Deep dyslexia—a case-study of connectionist neuropsychology	Cognitive Neuropsychology	586	1993	21	USA	N/A
17	Intensive remedial instruction for children with severe reading disabilities: immediate and long-term outcomes from two Instructional approaches	Journal of Learning Disabilities	550	2001	28	USA	1.333
18	Cognitive profiles of difficult-to-remediate and readily remediated poor readers: early intervention as a vehicle for distinguishing between cognitive and experiential deficits as basic causes of specific reading disability	Journal of Educational Psychology	537	1996	21	USA	N/A
19	Developmental dyslexia: the cerebellar deficit hypothesis	Trends in Neurosciences	511	2001	26	UK	16.475
20	Neural deficits in children with dyslexia ameliorated by behavioral remediation: evidence from functional MRI	Proceedings of the National Academy of Sciences of the United States of America	496	2003	28	USA	10.272
21	Developmental dyslexia—diagnostic approach based on 3 atypical reading-spelling patterns	Developmental Medicine and Child Neurology	492	1973	10	USA	N/A
22	The evidence for a temporal processing deficit linked to dyslexia: a review	Psychonomic Bulletin & Review	490	1995	19	Canada	N/A
23	Current concepts—dyslexia	New England Journal of Medicine	477	1998	21	USA	28.660
24	What is special about face recognition? Nineteen experiments on a person with visual object agnosia and dyslexia but normal face recognition	Journal of Cognitive Neuroscience	477	1997	20	Canada	4.844
25	Abnormal processing of visual motion in dyslexia revealed by functional brain imaging	Nature	477	1996	19	USA	N/A
26	Developmental dyslexia: specific phonological deficit or general sensorimotor dysfunction?	Current Opinion in Neurobiology	469	2003	26	France	9.727
27	Persistence of dyslexics phonological awareness deficits	Developmental Psychology	467	1992	16	Canada	N/A
28	Dyslexia (specific reading disability)	Biological Psychiatry	461	2005	29	USA	6.779
29	Evidence that dyslexia may represent the lower tail of a normal-distribution of reading-ability	New England Journal of Medicine	459	1992	16	USA	N/A
30	Brain morphology in developmental dyslexia and attention-deficit disorder hyperactivity	Archives of Neurology	454	1990	15	USA	N/A
31	Characteristics of developmental dyslexia in a regular writing system	Applied Psycholinguistics	443	1993	16	Austria	N/A
32	Quantitative trait locus for reading-disability on chromosome-6	Science	436	1994	16	USA	N/A
33	Functional connectivity of the angular gyrus in normal reading and dyslexia	Proceedings of the National Academy of Sciences of the United States of America	432	1998	19	USA	9.821
34	The non-word reading deficit in developmental dyslexia—a review	Reading Research Quarterly	432	1992	15	UK	N/A
35	Is developmental dyslexia a disconnection syndrome? Evidence from pet scanning	Brain	412	1996	16	UK	N/A
36	Explicit and implicit processing of words and pseudowords by adult developmental dyslexics—a search for wernicke's wortschatz?	Brain	403	1999	18	UK	7.374
37	A temporal sampling framework for developmental dyslexia	Trends in Cognitive Sciences	401	2011	40	UK	12.586
38	Lesioning an attractor network—investigations of acquired dyslexia	Psychological Review	401	1991	13	Canada	N/A
39	Developmental dyslexia: the visual attention span deficit hypothesis	Cognition	399	2007	29	France	3.831
40	The neurological basis of developmental dyslexia—an overview and working hypothesis	Brain	398	2000	19	Canada	7.303
41	Developmental lag vs. deficit models of reading disability: a longitudinal, individual growth curves analysis	Journal of Educational Psychology	397	1996	16	USA	N/A
42	Word-form dyslexia	Brain	396	1980	10	UK	N/A
43	Theoretical links among naming speed, precise timing mechanisms and orthographic skill in dyslexia	Reading and Writing	391	1993	14	Canada	N/A
44	Cytoarchitectonic abnormalities in developmental dyslexia—case-study	Annals of Neurology	391	1979	9	USA	N/A
45	Cognitive profiles of reading-disability—comparisons of discrepancy and low achievement definitions	Journal of Educational Psychology	388	1994	14	USA	N/A
46	Neuropsychological analyses of comorbidity between reading disability and attention deficit hyperactivity disorder: in search of the common deficit	Developmental Neuropsychology	387	2005	24	USA	2.443
47	Susceptibility loci for distinct components of developmental dyslexia on chromosomes 6 and 15	American Journal of Human Genetics	376	1997	16	USA	10.244
48	The impact of orthographic consistency on dyslexia: a German-English comparison	Cognition	373	1997	16	Austria	2.973
49	Functional neuroimaging studies of reading and reading disability (developmental dyslexia)	Mental Retardation and Developmental Disabilities Research Reviews	358	2000	17	USA	0.800
50	Phonemic deficits in developmental dyslexia	Psychological Research-Psychologische Forschung	348	1981	9	UK	N/A
51	Is preschool language impairment a risk factor for dyslexia in adolescence?	Journal of Child Psychology and Psychiatry and Allied Disciplines	342	2000	16	UK	2.940
52	Dyslexia: a deficit in visuo-spatial attention, not in phonological processing	Trends in Cognitive Sciences	340	2010	31	Australia	9.686
53	Are specific language impairment and dyslexia distinct disorders?	Journal of Speech Language and Hearing Research	336	2005	21	USA	1.725
54	Impaired processing of rapid stimulus sequences in dyslexia	Trends in Cognitive Sciences	336	2001	17	Finland	11.606
55	Amplitude envelope onsets and developmental dyslexia: a new hypothesis	Proceedings of the National Academy of Sciences of the United States of America	329	2002	17	UK	10.700
56	Rapid automatized naming (ran) and reading fluency: implications for understanding and treatment of reading disabilities	Annual Review of Psychology	328	2012	36	USA	15.265
57	Automaticity—a new framework for dyslexia research	Cognition	319	1990	10	UK	N/A
58	Family risk of dyslexia is continuous: individual differences in the precursors of reading skill	Child Development	310	2003	17	UK	3.324
59	Predicting dyslexia from kindergarten: the importance of distinctness of phonological representations of lexical items	Reading Research Quarterly	309	1998	13	Denmark	1.541
60	Sensitivity to dynamic auditory and visual stimuli predicts non-word reading ability in both dyslexic and normal readers	Current Biology	303	1998	13	UK	7.855
61	Dyslexia in children and young-adults–3 independent neuropsychological syndromes	Developmental Medicine and Child Neurology	303	1975	7	USA	N/A
62	Neural systems for compensation and persistence: young adult outcome of childhood reading disability	Biological Psychiatry	301	2003	17	USA	6.039
63	On the bases of two subtypes of development dyslexia	Cognition	299	1996	12	USA	N/A
64	Relations among speech, language, and reading disorders	Annual Review of Psychology	295	2009	25	USA	22.750
65	Neurobiological studies of reading and reading disability	Journal of Communication Disorders	295	2001	15	USA	0.640
66	Word-recognition skills of adults with childhood diagnoses of dyslexia	Developmental Psychology	295	1990	10	Canada	N/A
67	Biological abnormality of impaired reading is constrained by culture	Nature	294	2004	17	China	32.182
68	Developmental dyslexia	Lancet	293	2004	17	France	21.713
69	Subtypes of reading disability: variability around a phonological core	Journal of Educational Psychology	291	1998	13	USA	1.595
70	Understanding Chinese developmental dyslexia: morphological awareness as a core cognitive construct	Journal of Educational Psychology	290	2006	19	China	2.025
71	On the specifics of specific reading disability and specific language impairment	Journal of Child Psychology and Psychiatry and Allied Disciplines	286	2000	14	UK	2.940
72	DCDC2 is associated with reading disability and modulates neuronal development in the brain	Proceedings of the National Academy of Sciences of the United States of America	285	2005	18	USA	10.231
73	Dyslexia: a new synergy between education and cognitive neuroscience	Science	284	2009	24	USA	29.747
74	Toward a definition of dyslexia	Annals of Dyslexia	284	1995	11	USA	N/A
75	Estimating the risk of future reading difficulties in kindergarten children: a research-based model and its clinical implementation	Language Speech and Hearing Services in Schools	282	2001	14	USA	0.558
76	Early reading development in children at family risk for dyslexia	Child Development	281	2001	14	USA	2.583
77	Developmental dyslexia	Lancet	279	2012	31	USA	39.060
78	Functional abnormalities in the dyslexic brain: a quantitative meta-analysis of neuroimaging studies	Human Brain Mapping	278	2009	23	Austria	6.256
79	Contrast sensitivity and coherent motion detection measured at photopic luminance levels in dyslexics and controls	Vision Research	276	1995	11	UK	N/A
80	Evidence for aberrant auditory anatomy in developmental dyslexia	Proceedings of the National Academy of Sciences of the United States of America	275	1994	10	USA	N/A
81	Psychiatric comorbidity in children and adolescents with reading disability	Journal of Child Psychology and Psychiatry	274	2000	13	USA	2.940
82	Persistence of dyslexia: the Connecticut longitudinal study at adolescence	Pediatrics	270	1999	12	USA	3.487
83	Functional and morphometric brain dissociation between dyslexia and reading ability	Proceedings of the National Academy of Sciences of The United States of America	269	2007	19	USA	9.598
84	Dyslexia-specific brain activation profile becomes normal following successful remedial training	Neurology	268	2002	14	USA	5.340
85	A candidate gene for developmental dyslexia encodes a nuclear tetratricopeptide repeat domain protein dynamically regulated in brain	Proceedings of the National Academy of Sciences of The United States of America	264	2003	15	Finland	10.272
86	Neural systems predicting long-term outcome in dyslexia	Proceedings of the National Academy of Sciences of the United States of America	263	2011	26	USA	9.681
87	Developmental dyslexia in women—neuropathological findings in 3 patients	Annals of Neurology	262	1990	8	USA	N/A
88	MRI evaluation of the size and symmetry of the planum-temporale in adolescents with developmental dyslexia	Brain and Language	260	1990	8	Norway	N/A
89	Paying attention to reading: the neurobiology of reading and dyslexia	Development and Psychopathology	257	2008	20	USA	5.483
90	Impaired visual word processing in dyslexia revealed with magnetoencephalography	Annals of Neurology	257	1996	10	Finland	N/A
91	Developmental dyslexia in different languages: language-specific or universal?	Journal of Experimental Child Psychology	253	2003	14	France	1.482
92	The magnocellular deficit theory of dyslexia: the evidence from contrast sensitivity	Vision Research	253	2000	12	USA	2.000
93	Disrupted neural responses to phonological and orthographic processing in dyslexic children: an fMRI study	Neuroreport	252	2001	13	USA	2.374
94	The angular gyrus in developmental dyslexia: task-specific differences in functional connectivity within posterior cortex	Psychological Science	251	2000	12	USA	2.565
95	Phonological awareness deficits in developmental dyslexia and the phonological representations hypothesis	Journal of Experimental Child Psychology	250	1997	10	UK	1.333
96	Surface dyslexia	Quarterly Journal of Experimental Psychology Section A-Human Experimental Psychology	250	1983	7	UK	N/A
97	Predicting dyslexia at 8 years of age using neonatal brain responses	Brain and Language	248	2000	12	USA	1.473
98	Developmental dyslexia: genetic dissection of a complex cognitive trait	Nature Reviews Neuroscience	247	2002	13	UK	24.047
99	Semantic access dyslexia	Brain	246	1979	6	UK	N/A
100	Precursors of literacy delay among children at genetic risk of dyslexia	Journal of Child Psychology and Psychiatry	245	2000	12	UK	2.940

*USA, the United States of America; UK, the United Kingdom.*

* *“N/A” was assigned when the journal impact factor was not available or had not been assigned in the year of publication*.

### Journals

The different journals of the 100 top-cited studies on dyslexia and their associated impact factors are listed in Table 2. The 100 top-cited studies on dyslexia were published in 50 different journals, with the top three in frequency being “Proceedings of the National Academy of Sciences of the United States of America” (*n* = 10), “Brain” (*n* = 6), and “Journal of Educational Psychology” (*n* = 6).

**Table 2:** Journals of the 100 top-cited studies on dyslexia.

**Journal**	**Total citation times**	**Number of studies**	**Average citation times per study**	Impact factor (2019)^*^
Proceedings of the National Academy of Sciences of the United States of America	3,879	10	388	9.412
Brain	2,687	6	448	11.337
Journal of Educational Psychology	2,951	6	492	5.028
Cognition	2,025	5	405	3.294
Annals of Neurology	1,865	4	466	9.037
Biological Psychiatry	1,393	3	464	12.095
Journal of Child Psychology and Psychiatry	1,878	3	626	7.035
Science	1,385	3	462	41.846
Trends in Cognitive Sciences	1,077	3	359	15.218
Annals of Dyslexia	1,147	2	574	1.595
Annual Review of Psychology	623	2	312	18.111
Brain and Language	508	2	254	2.339
Child Development	591	2	296	4.891
Developmental Medicine and Child Neurology	795	2	398	4.406
Developmental Psychology	762	2	381	3.063
Journal of Child Psychology and Psychiatry and Allied Disciplines	628	2	314	7.035
Journal of Experimental Child Psychology	503	2	252	2.301
Lancet	572	2	286	60.390
Nature	771	2	386	42.779
New England Journal of Medicine	936	2	468	74.699
Psychological Bulletin	2,154	2	1,077	20.838
Psychological Review	999	2	500	6.844
Reading Research Quarterly	741	2	371	3.543
Trends In Neurosciences	1,235	2	618	12.891
Vision Research	529	2	265	2.610
American Journal of Human Genetics	376	1	376	10.502
Applied Psycholinguistics	443	1	443	1.412
Archives of Neurology	454	1	454	7.419
Cognitive Neuropsychology	586	1	586	2.396
Current Biology	303	1	303	9.601
Current Opinion in Neurobiology	469	1	469	6.263
Development and Psychopathology	257	1	257	3.385
Developmental Neuropsychology	387	1	387	1.477
Human Brain Mapping	278	1	278	4.421
Journal of Cognitive Neuroscience	477	1	477	3.105
Journal of Communication Disorders	295	1	295	1.315
Journal of Learning Disabilities	550	1	550	2.144
Journal of Speech Language and Hearing Research	336	1	336	1.873
Language Speech and Hearing Services in Schools	282	1	282	1.740
Mental Retardation and Developmental Disabilities Research Reviews	358	1	358	3.800
Nature Reviews Neuroscience	247	1	247	33.654
Neurology	268	1	268	8.770
Neuropsychologia	970	1	970	2.652
Neuroreport	252	1	252	1.394
Pediatrics	270	1	270	5.359
Psychological Research-Psychologische Forschung	348	1	348	2.419
Psychological Science	251	1	251	5.367
Psychonomic Bulletin & Review	490	1	490	3.910
Quarterly Journal of Experimental Psychology Section A-Human Experimental Psychology	250	1	250	2.449
Reading and Writing	391	1	391	1.445

* *Impact factors were extracted from the journal citation report of 2019. If the journal did not have an impact factor for 2019, its impact factor was expressed for the last year*.

The journal with the highest total citation count was “Proceedings of the National Academy of Sciences of the United States of America.” However, the highest average citation count per study belonged to the journal “Psychological Bulletin.” The journal impact factors of the 100 top-cited studies on dyslexia ranged from 1.315 to 74.699. Of the 100 top-cited studies, 29 were published in a journal with an impact factor greater than 10. The standard “CNS” journals, with the exception of “Cell,” “Nature,” and “Science” published 2 and 3 studies, respectively. Regarding the top four medical journals, while the “New England Journal of Medicine” and “Lancet” published 2 studies each, no top-cited study was published by the “Journal of the American Medical Association” or the “British Medical Journal.”

### Language and Year of Publication

The 100 top-cited studies on dyslexia were all published in English and were published between 1973 [by Boder et al. (23)] and 2012 [by Norton et al. (24) and Peterson et al. (25)] (Table 3). The most productive years were 2000, 2001 and 2003, with 9, 8 and 8 published articles, respectively. The year of 2003 had the most total citations with a total count of 3,788 and an average citation count per study of 474.

**Table 3:** Publication year of the 100 top-cited studies on dyslexia.

**Year**	**Number of studies**	**Total citation times**	**Average citation times**
2000	9	2,655	295
2001	8	3,172	397
2003	8	3,788	474
1996	6	2,379	397
1998	6	2,410	402
1990	5	1,590	318
1997	5	2,200	440
2005	5	2,925	585
1993	4	2,055	514
1999	4	2,319	580
2002	4	1,475	369
2004	4	2,644	661
1992	3	1,358	453
1994	3	1,099	366
1995	3	1,050	350
2009	3	857	286
1979	2	637	319
1991	2	1,069	535
2007	2	668	334
2011	2	664	332
2012	2	607	304
1973	1	492	492
1975	1	303	303
1976	1	970	970
1980	1	396	396
1981	1	348	348
1983	1	250	250
1985	1	955	955
2006	1	290	290
2008	1	257	257
2010	1	340	340

### Countries and Authors

Eleven countries contributed articles to the 100 top-cited studies on dyslexia (Table 4). Most of the articles were from the USA (*n* = 53), United Kingdom (*n* = 21), Canada (*n* = 7), and France (*n* = 6). In addition, the USA had the highest total citation count (23,129), and Italy had the highest average citation count per study (665).

**Table 4:** Countries of the 100 top-cited studies on dyslexia.

Country^*^	**Number of studies**	**Total citation times**	**Average citation times**
USA	53	23,129	436
UK	21	7,728	368
Canada	7	2,919	417
France	6	3,702	617
Austria	3	1,094	365
Finland	3	857	286
Australia	2	975	488
China	2	584	292
Denmark	1	309	309
Italy	1	665	665
Norway	1	260	260

*USA, the United States of America; UK, the United Kingdom.*

* *The country distribution of 100 top-cited studies on dyslexia research were extracted from the corresponding author*.

As shown in Table 5, there were 18 first-authors and 13 last-authors who published more than one of the 100 top-cited studies on dyslexia. Among them, Shaywitz SE published the most top 100 articles (*n* = 7) on dyslexia as the first author, followed by Galaburda AM (*n* = 3) and Pugh KR (*n* = 3). And for the last author, 8 studies of the 100 top-cited studies on dyslexia research were published by Shaywitz BA who was the most productive.

**Table 5:** Authors with at least two first-author or last-author publications in the 100 top-cited studies on dyslexia.

**First-author**		**Last-author**	
**Name**	**Number of studies**	**Name**	**Number of studies**
Shaywitz SE	7	Shaywitz BA	8
Galaburda AM	3	Frith U	5
Pugh KR	3	Gabrieli JDE	5
Bruck M	2	Gore JC	4
Catts HW	2	Shallice T	4
Goswami U	2	Goswami U	3
Hoeft F	2	Snowling MJ	3
Lyon GR	2	Bruck M	2
Nicolson RI	2	Defries JC	2
Pennington BF	2	Galaburda AM	2
Paulesu E	2	Pennington BF	2
Ramus F	2	Wimmer H	2
Snowling MJ	2	Wolf M	2
Temple E	2		
Vellutino FR	2		
Warrington EK	2		
Willcutt EG	2		
Ziegler JC	2		

### Publication Type and Web of Science Subject Categories

As shown in Table 6, there were 71 studies in the form of an original research article, 28 studies in the form of a review article, and one study in the form of an editorial material publication. The total citation counts for each publication type were 27,812, 13,899, and 511, respectively. Although the type of original research article had the highest total citation count, it had the lowest average citation count per study. In addition, a total of 12 Web of Science subject categories were extracted. Among them, “Psychology” was the most frequent category associated with studies [35], followed by “Clinical Neurology” [15], and “Multidisciplinary Sciences” [15], “Neurosciences” [12], and “Education” [6]. Consistent with the number of studies, the subject categories of “Psychology” and “Clinical Neurology” also had the highest total citation counts (15,683 and 6,427, respectively). The “Behavioral Sciences” subject category had the highest average citation count.

**Table 6:** Type of study and subject categories for the 100 top-cited studies on dyslexia.

**Variable**	**Number of studies**	**Total citation times**	**Average citation times per study**
**Type of study**			
Article	71	27,812	392
Review	28	13,899	496
Editorial Material	1	511	511
Web of Science categories^*^			
Psychology	35	15,683	448
Clinical Neurology	15	6,427	428
Multidisciplinary Sciences	15	6,035	402
Neurosciences	12	4,880	407
Education	6	2,829	472
Audiology & Speech-Language Pathology	5	1,421	284
Behavioral Sciences	4	2,047	512
Medicine	4	1,508	377
Biochemistry & Molecular Biology	1	303	303
Genetics & Heredity	1	376	376
Linguistics	1	443	443
Pediatrics	1	270	270

* *Web of Science subject categories were extracted from the Web of Science. If one article was listed in more than one category, the first category was used for data analysis*.

## Discussion

Although retrospective bibliometric approach has been conducted in many other diseases, to our knowledge, no citation analyses have examined publications on dyslexia. Therefore, this study is the first comprehensive analysis summarizing several features of the most influential studies on dyslexia. It has been suggested that a highly cited study can be considered as a milestone study in a related field and has the potential to generate meaningful changes in clinical practice (26). We believe that the present analysis of the 100 top-cited studies on dyslexia may be beneficial to the research community for the following reasons. First, the present study not only provides a historical projection of the scientific progress with regards to dyslexia research, but it also shows associated research trends and gaps in the field (27). Second, our findings provide critical quantitative information about how both the classic studies and recent advancements in the field have improved our understanding of dyslexia (28). Third, the present analysis may help journal editors, funding agencies, and reviewers critically evaluate studies and funding applications (28).

Our analysis discovered that the 100 top-cited studies on dyslexia were published in 50 different journals. This may reflect the fact that the 100 top-cited studies on dyslexia were very multidisciplinary in nature, unlike the top studies of other fields (e.g., psoriatic arthritis) where there is a more inherent researcher bias for journal selection (29). Of the 100 top-cited studies, 29 were published in a journal with an impact factor >10, and 62 studies were published in journal with an impact factor >5. However, there were only five studies published in the standard “CNS” journals and only four published in the top four medical journals, which suggests that most dyslexia researchers are more inclined to choose the most influential journals in their respective professional fields when submitting articles (30). This is in marked contrast with some other fields (e.g. vaccines), where the majority of top-cited articles are published in either the standard “CNS” journals or in the top four medical journals (15). Several other factors, such as the review turnaround time, likelihood of manuscript acceptance, publication costs, journal publication frequency, will all invariably also affect a researcher's journal selection (13, 20).

According to the results of our analysis, nearly 80% of the 100 top-cited studies on dyslexia were published between 1990 and 2005, and the years of 2000 was found to have the most publications. The increase of landmark publications between 1990 and 2005 might reflect an increase in the interest in dyslexia research or that researchers had made some important scientific breakthroughs during this time period. All the top-cited studies on dyslexia were published in English, likely because English is the most commonly used language for knowledge dissemination in the world.

The top countries with regards to total citation count and number of papers in the top 100 list were the USA (*n* = 53) and United Kingdom (*n* = 21), which accounted for ~75% of the 100 top-cited studies. The USA published the most studies from the list, and this is probably because some of the world's top research centers are located in the USA and likely also the USA receives more research funding (31). Furthermore, the most prolific first-author (Shaywitz SE) and last-author (Shaywitz BA) were also from the USA. It is also worth mentioning that China had two studies on the top 100 list, which attests to the improvement of our national scientific research community with regards to knowledge dissemination.

In the present study, there were more original research articles (*n* = 71) than review articles (*n* = 28), but the latter had higher average citation counts per study. These results indicate that even though researchers pay significant attention to new findings on dyslexia, they regularly use information from review articles to convey relevant points in their own papers. We found that “Psychology” was the most frequent subject category associated with the top 100 articles, which indicates that researchers have been working to find effective treatments for people with dyslexia and that research in this field will continue to progress.

Like with other bibliometric analyses, there are some study limitations that should be highlighted. First, the 100 top-cited studies were extracted from the Web of Science Core Collection, which might have excluded some top-cited studies from other databases, such as Scopus and Google Scholar. Second, there was no citation data for recently published studies. Third, self-citations might have substantially influenced the results of the citation analysis. Moreover, this was a cross-sectional study, which implies that the identified 100 top-cited studies could change in the future. Despite these limitations, this descriptive bibliometric study could contribute new information about the scientific interest in dyslexia.

In conclusion, the present analysis is the first analysis to recognize the 100 top-cited studies in the field of dyslexia. This analysis provides a better understanding on dyslexia and may help doctors, researchers, and stakeholders to achieve a more comprehensive understanding of classic studies, new discoveries, and trends regarding this research field. As new data continue to emerge, this bibliometric analysis will become an important quantitative instrument to ascertain the overall direction of a given field, thus promoting ideas for future investigation.

## Data Availability Statement

The original contributions presented in the study are included in the article/Supplementary Material, further inquiries can be directed to the corresponding author/s.

## Author Contributions

YZ and HF designed the study. SZ and YZ acquired the data and performed statistical analyses. SZ, YZ, and HF drafted the manuscript. All authors critically revised the article and approved the final version of the manuscript.

## Conflict of Interest

The authors declare that the research was conducted in the absence of any commercial or financial relationships that could be construed as a potential conflict of interest.
