# Supplementary material for: The 100 Top-Cited Studies on Dyslexia Research: A Bibliometric Analysis
Source: Front Psychiatry. 2021 Jul 22;12:714627. doi: 10.3389/fpsyt.2021.714627 (PMC8339432; doi:10.3389/fpsyt.2021.714627)
Supplement: Supplementary file 1 [file Table_1.DOCX]

**Supplementary Table 1.** The 100 top-cited studies on dyslexia based on mean citation per year.

| Ranking | Title | Journal | Total citation | Publication Year | Mean citation per year |
| --- | --- | --- | --- | --- | --- |
| 1 | Reading acquisition, developmental dyslexia, and skilled reading across languages: a psycholinguistic grain size theory | Psychological Bulletin | 1472 | 2005 | 92 |
| 2 | Specific reading disability (dyslexia): what have we learned in the past four decades? | Journal of Child Psychology and Psychiatry | 1368 | 2004 | 80 |
| 3 | Understanding normal and impaired word reading: computational principles in quasi-regular domains | Psychological Review | 1757 | 1996 | 70 |
| 4 | A definition of dyslexia | Annals of Dyslexia | 872 | 2003 | 48 |
| 5 | The double-deficit hypothesis for the developmental dyslexias | Journal of Educational Psychology | 1052 | 1999 | 48 |
| 6 | Theories of developmental dyslexia: insights from a multiple case study of dyslexic adults | Brain | 838 | 2003 | 47 |
| 7 | Developmental dyslexia and specific language impairment: same or different? | Psychological Bulletin | 703 | 2004 | 41 |
| 8 | A temporal sampling framework for developmental dyslexia | Trends in Cognitive Sciences | 404 | 2011 | 40 |
| 9 | Rapid automatized naming (ran) and reading fluency: implications for understanding and treatment of reading disabilities | Annual Review of Psychology | 332 | 2012 | 37 |
| 10 | Dyslexia: cultural diversity and biological unity | Science | 666 | 2001 | 33 |
| 11 | Disruption of posterior brain systems for reading in children with developmental dyslexia | Biological Psychiatry | 632 | 2002 | 33 |
| 12 | Developmental dyslexia | Lancet | 287 | 2012 | 32 |
| 13 | Dyslexia: a deficit in visuo-spatial attention, not in phonological processing | Trends in Cognitive Sciences | 343 | 2010 | 31 |
| 14 | Sensory theories of developmental dyslexia: three challenges for research | Nature Reviews Neuroscience | 183 | 2015 | 31 |
| 15 | To see but not to read; the magnocellular theory of dyslexia | Trends in Neurosciences | 730 | 1997 | 30 |
| 16 | Dyslexia (specific reading disability) | Biological Psychiatry | 463 | 2005 | 29 |
| 17 | Developmental dyslexia: the visual attention span deficit hypothesis | Cognition | 403 | 2007 | 29 |
| 18 | Action video games make dyslexic children read better | Current Biology | 227 | 2013 | 28 |
| 19 | Neural deficits in children with dyslexia ameliorated by behavioral remediation: evidence from functional MRI | Proceedings of the National Academy of Sciences of the United States of America | 498 | 2003 | 28 |
| 20 | Intensive remedial instruction for children with severe reading disabilities: immediate and long-term outcomes from two instructional approaches | Journal of Learning Disabilities | 552 | 2001 | 28 |
| 21 | Phonology, reading acquisition, and dyslexia: insights from connectionist models | Psychological Review | 601 | 1999 | 27 |
| 22 | Developmental dyslexia | Annual Review of Clinical Psychology | 161 | 2015 | 27 |
| 23 | Developmental dyslexia - 4 consecutive patients with cortical anomalies | Annals of Neurology | 958 | 1985 | 27 |
| 24 | Neural systems predicting long-term outcome in dyslexia | Proceedings of the National Academy of Sciences of the United States of America | 265 | 2011 | 27 |
| 25 | Developmental dyslexia: specific phonological deficit or general sensorimotor dysfunction? | Current Opinion in Neurobiology | 474 | 2003 | 26 |
| 26 | Functional disruption in the organization of the brain for reading in dyslexia | Proceedings of the National Academy of Sciences of the United States of America | 599 | 1998 | 26 |
| 27 | Developmental dyslexia: the cerebellar deficit hypothesis | Trends in Neurosciences | 513 | 2001 | 26 |
| 28 | Intact but less accessible phonetic representations in adults with dyslexia | Science | 201 | 2013 | 25 |
| 29 | Relations among speech, language, and reading disorders | Annual Review of Psychology | 297 | 2009 | 25 |
| 30 | Neuropsychological analyses of comorbidity between reading disability and attention deficit hyperactivity disorder: in search of the common deficit | Developmental Neuropsychology | 389 | 2005 | 24 |
| 31 | Dyslexia: a new synergy between education and cognitive neuroscience | Science | 287 | 2009 | 24 |
| 32 | Predictors of developmental dyslexia in European orthographies with varying complexity | Journal of Child Psychology and Psychiatry | 190 | 2013 | 24 |
| 33 | Functional abnormalities in the dyslexic brain: a quantitative meta-analysis of neuroimaging studies | Human Brain Mapping | 279 | 2009 | 23 |
| 34 | Early detection of dyslexia risk: development of brief, teacher-administered screens | Learning Disability Quarterly | 23 | 2020 | 23 |
| 35 | Varieties of developmental dyslexia | Cognition | 637 | 1993 | 23 |
| 36 | Physiological and anatomical evidence for a magnocellular defect in developmental dyslexia | Proceedings of the National Academy of Sciences of the United States of America | 670 | 1991 | 22 |
| 37 | Oral language deficits in familial dyslexia: a meta-analysis and review | Psychological Bulletin | 111 | 2016 | 22 |
| 38 | Rapid automatized naming (ran) - dyslexia differentiated from other learning-disabilities | Neuropsychologia | 972 | 1976 | 22 |
| 39 | Phonological deficits in specific language impairment and developmental dyslexia: towards a multidimensional model | Brain | 172 | 2013 | 22 |
| 40 | Cognitive profiles of difficult-to-remediate and readily remediated poor readers: early intervention as a vehicle for distinguishing between cognitive and experiential deficits as basic causes of specific reading disability | Journal of Educational Psychology | 537 | 1996 | 21 |
| 41 | Are specific language impairment and dyslexia distinct disorders? | Journal of Speech Language and Hearing Research | 338 | 2005 | 21 |
| 42 | Deep dyslexia - a case-study of connectionist neuropsychology | Cognitive Neuropsychology | 588 | 1993 | 21 |
| 43 | Current concepts - dyslexia | New England Journal of Medicine | 477 | 1998 | 21 |
| 44 | Meta-analyzing brain dysfunctions in dyslexic children and adults | Neuroimage | 207 | 2011 | 21 |
| 45 | What is special about face recognition? Nineteen experiments on a person with visual object agnosia and dyslexia but normal face recognition | Journal of Cognitive Neuroscience | 477 | 1997 | 20 |
| 46 | Paying attention to reading: the neurobiology of reading and dyslexia | Development and Psychopathology | 258 | 2008 | 20 |
| 47 | Understanding Chinese developmental dyslexia: morphological awareness as a core cognitive construct | Journal of Educational Psychology | 295 | 2006 | 20 |
| 48 | Functional and morphometric brain dissociation between dyslexia and reading ability | Proceedings of the National Academy of Sciences of the United States of America | 273 | 2007 | 20 |
| 49 | A qualitative and quantitative review of diffusion tensor imaging studies in reading and dyslexia | Neuroscience and Biobehavioral Reviews | 175 | 2012 | 19 |
| 50 | Neuroanatomy of developmental dyslexia: pitfalls and promise | Neuroscience and Biobehavioral Reviews | 58 | 2018 | 19 |
| 51 | Unstable representation of sound: a biological marker of dyslexia | Journal of Neuroscience | 154 | 2013 | 19 |
| 52 | Abnormal processing of visual motion in dyslexia revealed by functional brain imaging | Nature | 480 | 1996 | 19 |
| 53 | The neurological basis of developmental dyslexia - an overview and working hypothesis | Brain | 400 | 2000 | 19 |
| 54 | The evidence for a temporal processing deficit linked to dyslexia: a review | Psychonomic Bulletin & Review | 491 | 1995 | 19 |
| 55 | Functional connectivity of the angular gyrus in normal reading and dyslexia | Proceedings of the National Academy of Sciences of the United States of America | 433 | 1998 | 19 |
| 56 | A tractography study in dyslexia: neuroanatomic correlates of orthographic, phonological and speech processing | Brain | 169 | 2012 | 19 |
| 57 | Music, rhythm, rise time perception and developmental dyslexia: perception of musical meter predicts reading and phonology | Cortex | 187 | 2011 | 19 |
| 58 | Explicit and implicit processing of words and pseudowords by adult developmental dyslexics - a search for wernicke's wortschatz? | Brain | 403 | 1999 | 18 |
| 59 | DCDC2 is associated with reading disability and modulates neuronal development in the brain | Proceedings of the National Academy of Sciences of the United States of America | 286 | 2005 | 18 |
| 60 | Biological abnormality of impaired reading is constrained by culture | Nature | 298 | 2004 | 18 |
| 61 | The current status of the magnocellular theory of developmental dyslexia | Neuropsychologia | 35 | 2019 | 18 |
| 62 | Neurobiology of dyslexia | Current Opinion in Neurobiology | 105 | 2015 | 18 |
| 63 | Amplitude envelope onsets and developmental dyslexia: a new hypothesis | Proceedings of the National Academy Of Sciences of the United States of America | 332 | 2002 | 17 |
| 64 | Comorbidity between reading disability and math disability: concurrent psychopathology, functional impairment, and neuropsychological functioning | Journal of Learning Disabilities | 139 | 2013 | 17 |
| 65 | Developmental dyslexia | Lancet | 295 | 2004 | 17 |
| 66 | Family risk of dyslexia is continuous: individual differences in the precursors of reading skill | Child Development | 311 | 2003 | 17 |
| 67 | Extra-large letter spacing improves reading in dyslexia | Proceedings of the National Academy of Sciences of the United States of America | 155 | 2012 | 17 |
| 68 | Functional neuroimaging studies of reading and reading disability (developmental dyslexia) | Mental Retardation and Developmental Disabilities Research Reviews | 358 | 2000 | 17 |
| 69 | Dyslexia and dyscalculia: two learning disorders with different cognitive profiles | Journal of Experimental Child Psychology | 203 | 2009 | 17 |
| 70 | Effectiveness of treatment approaches for children and adolescents with reading disabilities: a meta-analysis of randomized controlled trials | Plos One | 118 | 2014 | 17 |
| 71 | Impaired processing of rapid stimulus sequences in dyslexia | Trends in Cognitive Sciences | 337 | 2001 | 17 |
| 72 | Neural systems for compensation and persistence: young adult outcome of childhood reading disability | Biological Psychiatry | 302 | 2003 | 17 |
| 73 | Is developmental dyslexia a disconnection syndrome? evidence from pet scanning | Brain | 412 | 1996 | 16 |
| 74 | Is preschool language impairment a risk factor for dyslexia in adolescence? | Journal of Child Psychology and Psychiatry and Allied Disciplines | 343 | 2000 | 16 |
| 75 | Persistence of dyslexics phonological awareness deficits | Developmental Psychology | 468 | 1992 | 16 |
| 76 | How the visual aspects can be crucial in reading acquisition: the intriguing case of crowding and developmental dyslexia | Journal of Vision | 96 | 2015 | 16 |
| 77 | Characteristics of developmental dyslexia in a regular writing system | Applied Psycholinguistics | 446 | 1993 | 16 |
| 78 | Evidence that dyslexia may represent the lower tail of a normal-distribution of reading-ability | New England Journal of Medicine | 461 | 1992 | 16 |
| 79 | Developmental lag versus deficit models of reading disability: a longitudinal, individual growth curves analysis | Journal of Educational Psychology | 397 | 1996 | 16 |
| 80 | Basic auditory processing deficits in dyslexia: systematic review of the behavioral and event-related potential/field evidence | Journal of Learning Disabilities | 127 | 2013 | 16 |
| 81 | Susceptibility loci for distinct components of developmental dyslexia on chromosomes 6 and 15 | American Journal of Human Genetics | 377 | 1997 | 16 |
| 82 | Music training increases phonological awareness and reading skills in developmental dyslexia: a randomized control trial | Plos One | 94 | 2015 | 16 |
| 83 | The impact of orthographic consistency on dyslexia: a German-English comparison | Cognition | 376 | 1997 | 16 |
| 84 | Multiple causal links between magnocellular-dorsal pathway deficit and developmental dyslexia | Cerebral Cortex | 78 | 2016 | 16 |
| 85 | A multiple deficit model of reading disability and attention-deficit/hyperactivity disorder: searching for shared cognitive deficits | Journal of Child Psychology and Psychiatry | 156 | 2011 | 16 |
| 86 | Deviant processing of letters and speech sounds as proximate cause of reading failure: a functional magnetic resonance imaging study of dyslexic children | Brain | 170 | 2010 | 15 |
| 87 | Multisensory spatial attention deficits are predictive of phonological decoding skills in developmental dyslexia | Journal of Cognitive Neuroscience | 169 | 2010 | 15 |
| 88 | Children with developmental dyslexia showed greater sleep disturbances than controls, including problems initiating and maintaining sleep | Acta Paediatrica | 76 | 2016 | 15 |
| 89 | From genes to behavior in developmental dyslexia | Nature Neuroscience | 228 | 2006 | 15 |
| 90 | Disruption of functional networks in dyslexia: a whole-brain, data-driven analysis of connectivity | Biological Psychiatry | 105 | 2014 | 15 |
| 91 | The nonword reading deficit in developmental dyslexia - a review | Reading Research Quarterly | 434 | 1992 | 15 |
| 92 | The axon guidance receptor gene robo1 is a candidate gene for developmental dyslexia | Plos Genetics | 239 | 2005 | 15 |
| 93 | Neurobiological studies of reading and reading disability | Journal of Communication Disorders | 298 | 2001 | 15 |
| 94 | Altered low-gamma sampling in auditory cortex accounts for the three main facets of dyslexia | Neuron | 148 | 2011 | 15 |
| 95 | A candidate gene for developmental dyslexia encodes a nuclear tetratricopeptide repeat domain protein dynamically regulated in brain | Proceedings of the National Academy of Sciences of the United States of America | 266 | 2003 | 15 |
| 96 | Brain morphology in developmental dyslexia and attention-deficit disorder hyperactivity | Archives of Neurology | 455 | 1990 | 15 |
| 97 | Strong evidence that kiaa0319 on chromosome 6p is a susceptibility gene for developmental dyslexia | American Journal of Human Genetics | 233 | 2005 | 15 |
| 98 | Structural abnormalities in the dyslexic brain: a meta-analysis of voxel-based morphometry studies | Human Brain Mapping | 115 | 2013 | 14 |
| 99 | Cognitive profiles of reading-disability - comparisons of discrepancy and low achievement definitions | Journal of Educational Psychology | 388 | 1994 | 14 |
| 100 | Developmental dyslexia in different languages: language-specific or universal? | Journal of Experimental Child Psychology | 256 | 2003 | 14 |

Notes: This exhaustive literature retrieval was conducted on June 20th, 2021.
